# Detection of early locomotor abnormalities in a *Drosophila* model of Alzheimer's disease

**DOI:** 10.1016/j.jneumeth.2011.01.026

**Published:** 2011-04-15

**Authors:** Thomas R. Jahn, Kai J. Kohlhoff, Michael Scott, Gian Gaetano Tartaglia, David A. Lomas, Christopher M. Dobson, Michele Vendruscolo, Damian C. Crowther

**Affiliations:** a Department of Genetics, University of Cambridge, UK; b Department of Chemistry, University of Cambridge, UK; c School of Medicine, Stanford University, USA; d Center for Genomic Regulation, Barcelona, Spain; e Cambridge Institute for Medical Research, University of Cambridge, UK

**Keywords:** Neurodegenerative disorders, *Drosophila*, Alzheimer's disease, Behavioural assay, Computer vision

## Abstract

Behavioural assays represent sensitive methods for detecting neuronal dysfunction in model organisms. A number of manual methods have been established for *Drosophila*, however these are time-consuming and generate parameter-poor phenotype descriptors. Here, we have developed an automated computer vision system to monitor accurately the three-dimensional locomotor trajectories of flies. This approach allows the quantitative description of fly trajectories, using small fly cohorts and short acquisition times. The application of this approach to a *Drosophila* model of Alzheimer's disease enables the early detection of progressive locomotor deficits and the quantitative assessment of phenotype severity. The approach can be widely applied to different disease models in a number of model organisms.

## Research highlights

► We present a computer vision system to monitor locomotion in flies in 3D. ► A *Drosophila* model of Alzheimer's disease has been explored. ► A fast and quantitative assessment of phenotype severity is possible. ► The approach can be widely applied to different disease models.

## 1 Introduction

Protein misfolding and aggregation underpin many disorders of ageing (Fontana et al., 2010), the most common example being Alzheimer's disease (AD) (Mucke, 2009; Thal et al., 2004). The molecular processes involved in protein aggregation have been studied extensively *in vitro* (Chiti and Dobson, 2006) and in transgenic animal models of neurodegenerative disorders (Feany and Bender, 2000; Fernandez-Funez et al., 2000; Iijima et al., 2004; Lu and Vogel, 2009; Luheshi et al., 2007; Luo et al., 1992; Wittmann et al., 2001). There is particular interest in sensitive and quantitative methods to measure neuronal dysfunction in invertebrate models such as *Drosophila melanogaster*, the common fruit fly. We describe here an automated three-dimensional tracking system that allows the rapid measurement of the locomotor behaviour of individual flies and the calculation of a range of quantitative parameters that describe accurately their movements. We show that this method detects very early abnormalities in a fly model of AD through a more sensitive phenotype analysis than conventional methods.

**Fig. 1:**
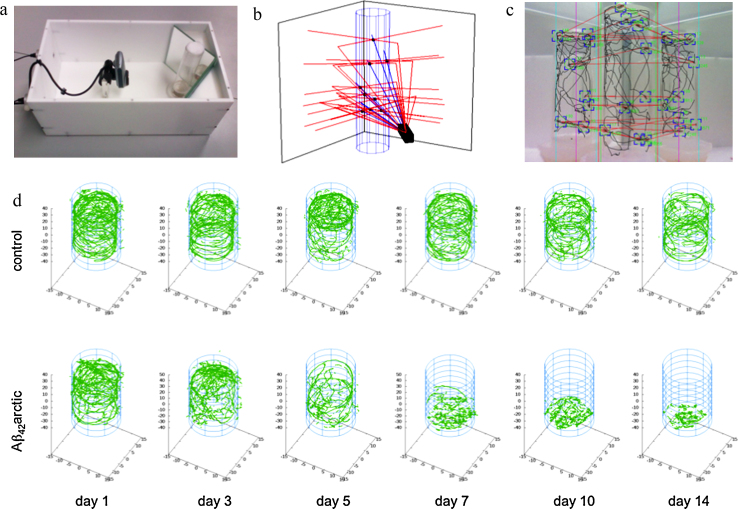
Experimental setup and examples of trajectories of individual flies. (a) Hardware configuration for the fly tracking chamber, consisting of a camera connected to a personal computer, two mirrors, two LED lights and a tube stage. (b) 3D reconstruction of the ray tracing calculations that allow images from two mirrors to triangulate with the direct image and hence accurately to locate and track the flies in the tube. (c) Camera view of a captured frame from the fly tracking software, indicating detected flies (blue boxes), triangulation vectors (red lines) as well as calculated trajectories (black lines). (d) Reconstruction of 3D trajectories of 10 control flies (top) and 10 flies expressing the Aβ_42_arctic peptide (bottom) within the measuring tube. Flies were aged at 29 °C and measured for a period of 15 s. (For interpretation of the references to color in this figure legend, the reader is referred to the web version of this article.)

The apparatus, named “iFly” (Fig. 1a), was designed to be as simple and robust as possible. It consists of a camera and two mirrors; the latter are positioned to generate simultaneously three images of each fly, allowing the three-dimensional coordinates to be recorded with high spatial (100 μm) and temporal (100–120 ms) resolution. We have developed computational procedures to detect and track individual flies and to parameterise their trajectories (Fig. 1b). This procedure allows the triangulation of each fly image in three dimensions and is defined as successful if two out of three constructed rays cross within 100 μm. This approach, contrary to other three-dimensional tracking systems, does not require multiple cameras and the challenging problem of synchronising multiple images (Fry et al., 2003; Grover et al., 2008). Acquisition of data at 7–10 frames per second using a conventional webcam allows the walking and climbing locomotor behaviour of individual flies to be tracked, while ignoring the motion of flying or falling flies (Fig. 1c). In contrast to ‘negative geotaxis’ assays (Gargano et al., 2005), we are able to automatically analyse the ‘geotaxis’ of numerous flies in an unbiased manner and are able to collect large series of locomotor data over time from a small cohort of flies.

To demonstrate the potential of the iFly method we examined flies that represent well-characterised models of both sporadic and familial AD (Crowther et al., 2005). We were able to obtain reproducible locomotor data using as few as ten female flies in clean, food-free tubes at the same time of the day and with identical illumination. We then acquired movies of locomotor behaviour over a period of 14 days where analysis reveals progressive locomotor deficits. Previous work has shown that flies expressing aggregation-prone forms of the amyloid β peptide (Aβ), the peptides that accumulate in the brains of patients with AD, can show gross locomotor abnormalities and experience premature death (Crowther et al., 2005). In the present studies, the phenotypic severity correlates well with the aggregation propensity of the expressed peptide. Thus, expression of the least aggregation-prone isoform, Aβ_40_, has no measureable consequences, while Aβ_42_, and even more dramatically E22G Aβ_42_ (the Arctic variant, Aβ_42_arctic), show clear phenotypes. The use of the fruit fly to model AD has many advantages in terms of speed and genetic flexibility; we now add to the power of the model system by presenting an automated locomotor assay that generates high-throughput quantitative locomotor data, allowing the early detection of AD-related phenotypes.

## 2 Materials and methods

### 2.1 iFly apparatus

The iFly apparatus is composed of an inexpensive off-the-shelf fixed-focus digital webcam linked to a standard Microsoft Windows personal computer. A tube of flies is placed in a chamber that is lit either through a frosted-plastic lid or by internal illumination. To enable three-dimensional tracking, two mirrors are placed at equal angles behind the tube to allow side images of the flies to be captured by the camera. In the configuration used in this study, the mirrors were placed at an angle of 39° to the wall of the chamber. A webcam with a horizontal field of view of 50° and VGA resolution was placed at a distance of 95 mm from the centre of the tube.

### 2.2 Fly rearing

Flies expressing different Aβ peptides were generated as described by Crowther et al. (2005) using the PhiC31 system (Bateman et al., 2006), allowing a comparable expression levels of different constructs. Elav-GAL4;UAS-Aβ flies were generated by crossing female elav-GAL4 flies with male UAS-Aβ or non-transgenic flies. Flies were reared in glass vials containing standard fly food (yeast, corn syrup and agar) at 29 °C, 60% humidity with a 12 h light–dark cycle. Newly eclosed female flies expressing one copy of Aβ_42_, Aβ_42_arctic (E22G) or the Aβ_40_ peptide, as well as flies with the same genetic background but without a transgene (control flies), were incubated at 29 °C in groups of 10 in 4-in. glass vials with new food provided every 2 days. For survival data, viable flies were counted every other day.

### 2.3 iFly measurements

Measuring tubes containing 10 flies were placed in the iFly apparatus at time *t*_0_ and videos were recorded for 90 s, with the tube briefly lifted and dropped back into the apparatus after *t*_1_ = *t*_0_ + 30 s and *t*_2_ = *t*_0_ + 60 s. In contrast to classical “negative geotaxis” assays, this mechanical stimulation was used to promote locomotor behaviour. All videos were processed using in-house developed software (KJK, TRJ, DAL, CMD, DCC and MV, described elsewhere). A pre-processing step, detected the gross movements that occur when the flies are tapped to the bottom of the tube, was used to determine *t*_0_, *t*_1_, and *t*_2_ automatically. This provided an unbiased start to each measurement period and assured that video fragments were acquired under identical lighting conditions. At times *t*_0_, *t*_1_, and *t*_2_ the flies have just fallen to the bottom of the tube and the video frames capture the full extent of their negatively geotactic walking movements. These trajectories of climbing behaviour were recorded on a hard disc for 15 s each in form of the time-stamped Cartesian coordinates of the derived fly positions in space. To assure that each trajectory belongs to a single fly, we have restricted the possible change in position between consecutive video frames to 2 mm. This restriction means that only walking behaviour is recorded as flight and falling result in more rapid movements.

### 2.4 Statistical analysis

The Cartesian coordinates for the locomotor behaviour were analyzed systematically to extract statistical descriptors of the fly populations. Descriptors include the velocity, the persistence length of the fly trajectories as well as the turn tightness. The persistence length for each fly trajectory was calculated as ratio of the distance between the start- and end-point of the trajectory (end-to-end distance) and the entire distance travelled by the fly in this trajectory (total distance). The turn tightness was calculated as a measure of the directionality of the path. Here, the trajectories were handled as a series of vectors. To describe the angular orientation between adjacent vectors, the dot product for vector pairs was calculated and the median of the dot products for all vectors is given as the turn tightness of a path. Statistical properties of these parameters were then used to discriminate healthy from sick flies with a very high level of confidence provided by the large amount of data made available through the automated data acquisition and analysis procedures. iFly analysis was performed in triplicate (i.e. three separate experiments with triplicates of each genotype measured in triplicate at each time point), showing a difference in experimental variation of less than 10%.

## 3 Results and discussion

Our initial goal was to test the extent to which the iFly locomotor analysis correlates with the conventional method of measuring dysfunction such as the longevity assay, the current “gold standard” test, and to define the sensitivity of the iFly method. A direct comparison of the trajectories of control non-transgenic flies and flies expressing the toxic Aβ_42_arctic variant (Fig. 1d) shows that control flies show no differences in behaviour throughout the first 14 days of life, while Aβ_42_arctic flies become significantly and progressively impaired from day 5 onwards. Qualitatively the trajectories become shorter, usually being terminated by the flies involved falling from the side of the tube, and the tracks are less unidirectional. After day 10 the Aβ_42_arctic flies are no longer able to climb the sides of the tubes.

**Fig. 2:**
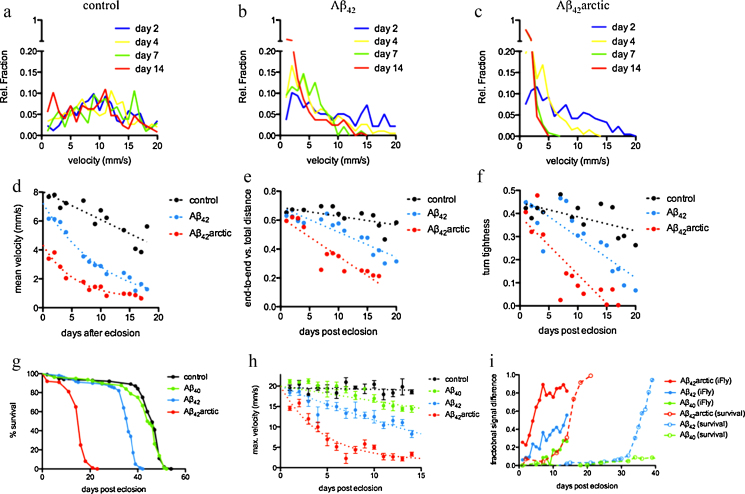
Locomotor analysis of *Drosophila* models of neurodegeneration. (a–c) Distribution of velocities for non-transgenic control flies (a), flies expressing the Aβ_42_ peptide (b), and flies expressing the more toxic Aβ_42_arctic peptide (c) are shown as a function of age (blue, yellow, green and red lines represent fly velocities after 2, 4, 7 and 14 days after eclosion, respectively). (d–f) Examples of quantitative parameters extracted from the computational analysis of fly trajectories, including the mean velocity (d), the ratio between end-to-end distance and total distance (e), and the calculated turn tightness of different fly paths, all clearly indicate differences between control flies (black), Aβ_42_ flies (light blue) and Aβ_42_arctic flies (red); dotted lines indicate the differential loss of locomotor function. (g–i) Comparison between a traditional longevity assay (g) and the iFly automated analysis of locomotor activity, parameterised by maximum velocity (h) for non-transgenic control flies (black) and flies expressing the non-toxic Aβ_40_ peptide (green), Aβ_42_ (blue) or the Aβ_42_arctic peptide (red). (i) The fractional signal difference between flies expressing Aβ and control flies clearly indicates the power of iFly (solid lines) to distinguish between these different fly lines early in life, well before any quantitative changes are apparent in a survival assay (dotted lines). (For interpretation of the references to color in this figure legend, the reader is referred to the web version of this article.)

Although these data by themselves reveal that iFly is able to track the movement of flies and permit a qualitative distinction between healthy and impaired flies, our aim in developing the method was to obtain quantitative descriptors of fly locomotion. From the trajectories we can first calculate the speed of fly locomotion by dividing the distance moved between frames by the elapsed time. Histograms of the speed defined in this way were plotted for control and Aβ-expressing flies of different ages (Fig. 2a–c). For control flies this distribution is rather broad and flat, ranging from 0 to 20 mm/s with a median value of approximately 10 mm/s (Fig. 2a); this distribution did not change significantly for control flies during the first 14 days of life. In contrast, during the same time span there was a clear change in the distribution of speed for flies expressing the Aβ_42_ peptide (Fig. 2b); while at day 2 these flies have a distribution similar to that of control flies, thereafter there is a statistically highly significant shift to lower velocities. This effect is even larger in flies expressing the more toxic Aβ_42_arctic variant (Fig. 2c) where the majority of flies are essentially immobile after 7 days. These findings are also illustrated by generating plots of the mean speed of flies of various ages and for each genotype (Fig. 2d), which show clear differences between Aβ_40_ (black), Aβ_42_ (blue) and Aβ_42_arctic (red) flies. As expected, there is a significant correlation between the decrease in the mean speed and the increase in the aggregation propensity of the different Aβ peptide variants. One major result of this study is to demonstrate that such differences can be quantified by the iFly approach from the first day of adult life, while measurements of the median lifespan require measurements for upwards of 30 days.

We next calculated a range of other parameters (see Section 2 for details) to define further the characterisation of the locomotor behaviour of the different groups of flies, including the persistence length (i.e. the ratio of end-to-end distance vs. total distance travelled for each fly trajectory, Fig. 2e) and the turn tightness (i.e. a measure of the non-linearity of the fly trajectories, Fig. 2f). We observe subtle behavioural changes already at very early stages in the fly life (Fig. 2g and h), and we found that the rate of decline of locomotor speed is correlated with, and therefore predictive of, the median survival (Fig. 2i), with statistically significant data from day 3 (*p* = 0.006). From these representative results, obtained for flies expressing different mutational variants of the Aβ peptide, we conclude that the descriptors of fly movement introduced here will enable the quantitative description of early signs of locomotor abnormalities and we anticipate that the method may result in the ability to detect the presence of particular toxic species in the brain or report damage to particular parts of the fly brain.

In summary, we have developed an automated method, which dramatically increases the sensitivity and throughput of *Drosophila* locomotor assays. Since the iFly method allows the initial signs of neuronal dysfunction to be detected much earlier than current assays, it enables us to significantly reduce the duration and costs of behavioural assays. The results of the present study suggest that similar automated locomotor analyses will assist the rapid detection of pathological behaviour in a variety of other model organisms. Finally, the automated and quantitative nature of iFly will facilitate large-scale screening studies that are sensitive to the early, and potentially reversible, stages of neurodegeneration.

## Author contributions

TRJ, KJK, DAL, CMD, MV and DCC designed research; TRJ conducted experiments; TRJ, KJK, MS, GGT and MV analyzed data; TRJ, DAL, CMD, MV and DCC wrote the paper.

## Competing interest statement

The authors declare no competing interest.
